# Is justice grounded? How expertise shapes conceptual representation of institutional concepts

**DOI:** 10.1007/s00426-021-01492-8

**Published:** 2021-03-07

**Authors:** Caterina Villani, Stefania D’Ascenzo, Anna M. Borghi, Corrado Roversi, Mariagrazia Benassi, Luisa Lugli

**Affiliations:** 1 Department of Philosophy and Communication, University of Bologna, Via Azzo Gardino, 23, 40122 Bologna, Italy; 2 Department of Dynamic and Clinical Psychology, and Health Studies, Sapienza University of Rome, Rome, Italy; 3 Institute of Cognitive Sciences and Technologies, Italian National Research Council, Rome, Italy; 4 Department of Legal Sciences, University of Bologna, Bologna, Italy; 5 Department of Psychology, University of Bologna, Bologna, Italy

## Abstract

Using abstract concepts is a hallmark of human cognition. While multiple kinds of abstract concepts exist, they so far have been conceived as a unitary kind in opposition to concrete ones. Here, we focus on Institutional concepts, like justice or norm, investigating their fine-grained differences with respect to other kinds of abstract and concrete concepts, and exploring whether their representation varies according to individual proficiency. Specifically, we asked experts and non-experts in the legal field to evaluate four kinds of concepts (i.e., institutional, theoretical, food, artefact) on 16 dimensions: abstractness-concreteness; imageability; contextual availability; familiarity; age of acquisition; modality of acquisition; social valence; social metacognition; arousal; valence; interoception; metacognition; perceptual modality strength; body-object interaction; mouth and hand involvement. Results showed that Institutional concepts rely more than other categories on linguistic/social and inner experiences and are primarily characterized by positive valence. In addition, a more subtle characterization of the institutional domain emerged: Pure-institutional concepts (e.g., parliament) were perceived as more similar to technical tools, while Meta-institutional concepts (e.g., validity) were characterized mainly by abstract components. Importantly, for what concerns individual proficiency, we found that the level of expertise affects conceptual representation. Only law-experts associated Institutional concepts with exteroceptive and emotional experiences, showing also a more grounded and situated representation of the two types of institutional concepts. Overall, our finding highlights the richness and flexibility of abstract concepts and suggests that they differ in the degree of embodiment and grounding. Implications of the results for current theories of conceptual representation and social institutions are discussed.

**Supplementary Information:**

The online version contains supplementary material available at 10.1007/s00426-021-01492-8.

## Introduction

Defining the meaning of words like “democracy” or “justice” might be much harder than defining that of more concrete words like “hat” or “cat.” And yet, well over 70% of the words we produce and understand are abstract in content (Lupyan & Winter, 2018). Abstract thought represents a sophisticated and important ability of our species: Providing an explanation of abstract concepts is, therefore, one of the key challenges for any theory of cognition.

Contemporary approaches widely agree on rejecting a marked dichotomy between abstract and concrete concepts (Wiemer-Hastings et al., 2001; Wiemer-Hastings & Xu, 2005; Barsalou, Dutriaux & Scheepers, 2018). Such a dichotomy posed important limitations: Concrete and abstract concepts were defined exclusively on their perceptual basis, respectively as denoting something that can either be directly experienced or not through our senses (i.e., Paivio, 1986; Brysbaert et al., 2014). As a consequence, defining abstract concepts negatively in terms of their lacking a physical and perceptible referent did not offer much insight into what they are.

According to embodied and grounded approaches, all concepts are intended as flexible entities, re-enacting and integrating relevant information of a given category in a situated context to support goal-oriented actions (Barsalou, 1999; Barsalou, 2008; Glenberg et al., 2013; Kiefer & Barsalou, 2013; Barsalou, Dutriaux, & Scheepers, 2018). Importantly, it is increasingly evident that the set of information retrieved by a concept might dynamically vary across contexts, ongoing tasks, and individual differences (for a review see Yee & Thompson-Schill, 2016). Despite the fact that all concepts are variable, conceptual flexibility might be more pronounced with abstract than with concrete concepts. Category members of abstract concepts are highly heterogeneous and refer to a broad range of situations compared to concrete concepts (e.g., “ethics” vs. “chair”). Abstract concepts also show a great intra-class diversity (e.g., Kiefer & Harpaintner, 2020), thereby their semantic content varies depending on the individual concepts (e.g., “logic" vs. “desire”).

One of the most fruitful lines of recent research consists in drawing fine-grained distinctions among concepts, beyond the two broad groups of abstract and concrete concepts.

Within concrete concepts, the distinction into sub-categories is widely supported by neuropsychological and neuroimaging evidence. Brain damage studies have reported category-specific semantic impairments (e.g., fruits, animals, tools) and focused particularly on the double dissociations between living and nonliving entities (Warrington & Shallice, 1984, for review see Capitani et al., 2003) or the corresponding distinction between natural kinds and artefacts (Keil, 1989). Recently, a lot of attention has been dedicated to the investigation of food, which can be considered as belonging both to natural objects and artifacts (e.g., Rumiati & Foroni, 2016). Moreover, there is compelling evidence that concrete concepts and action-related words are based on perceptual and motor information, leading to modality-specific activation of sensorimotor systems (Hauk, Johnsrude, & Pulvermüller, 2004; Glenberg & Gallese, 2012).

On the other hand, abstract concepts have often been considered as a unitary class, and their diversity has been almost completely overlooked in research on conceptual knowledge. This is likely due to the difficulty in identifying clear-cut categories within the abstract concepts’ domain, which includes very dissimilar kinds, each of which might evoke different types of experiences. While concrete concepts mainly activate perceptual properties of the words’ referents, abstract concepts, like “freedom” or “justice”, elicit higher proportions of complex and rich experiences, involving episodes and situational relations (Wiemer-Hastings & Xu, 2005), emotions (Kousta, Vigliocco, Vinson, Andrews, & Del Campo, 2011; Vigliocco et al., 2013), introspection (Barsalou & Wiemer-Hastings, 2005) and interoceptive states (Connell, Lynott & Banks, 2018). Because of the higher complexity of abstract concepts when compared to concrete ones, their representation could be more affected by linguistic, cultural, and individual variability (Borghi & Binkofski, 2014; Borghi, 2019; Barsalou, 1987). In recent years, interest in abstract concepts representation has yielded a lively debate (for special topics see Borghi et al., 2018a, b; Bolognesi & Steen, 2018), driven mainly by the observation that they do not seem to be suitable for grounding in perception and action systems. However, to gain a comprehensive and insightful understanding of this topic it is now becoming pivotal to focus more narrowly on specific domains of abstract concepts, and not treat them as an undifferentiated whole.

Recently, behavioral and neuroscientific studies are starting to explore the differences within abstract concepts. Several studies revealed peculiar kinds of abstract concepts grouped on the basis of their dominant features, such as emotional ones (i.e., characterized to engage bodily information; see Altarriba, Bauer, & Benvenuto, 1999; Altarriba & Bauer, 2004; Barca, Mazzuca, & Borghi, 2017, 2020; Mazzuca, Lugli, Benassi, Nicoletti, & Borghi, 2018; Ponari, Norbury, & Vigliocco, 2018; Lund, Sidhu, & Pexman, 2019), numbers and math-related concepts (i.e., strictly linked to hand effector and fingers counting habits; Fischer & Shaki, 2018; Fischer & Brugger, 2011; see also Ghio, Vaghi, & Tettamanti, 2013; Ghio, Haegert, Vaghi, & Tettamanti, 2018; and influences by congruent motion see Lugli et al., 2013, 2018; Anelli et al., 2014), social ones (Mellem, Jasmin, Peng, & Martin, 2016), moral/aesthetics concepts (Fingerhut & Prinz, 2018), theory of mind concepts (Desai, Reilly, & van Dam, 2018).

Overall, these studies suggest that the concrete-abstract dichotomy is an overly simplistic distinction, that their relations can be better represented in multidimensional spaces where some of their features overlap (Harpaintner, Trumpp, & Kiefer, 2018; Crutch et al., 2013; Villani et al., 2019), and that different kinds of abstract concepts exist. Some of them have received a great deal of attention, such as emotions and numbers, while others have not been considered yet.

Within this theoretical framework, in this study, we will explore a specific kind of concept for which both concrete and abstract components are relevant. Specifically, we will focus on institutional concepts, namely, concepts that connote either an institution or an institutional element, like “norm”, “parliament”, “contract”. We purposely use the term “institution” in a broad way, to include both basic concepts like “norm” and more formalized ones like “contract”, for a reason that will become apparent in what follows. In general, however, these concepts describe entities constituted by more or less formalized rules in a social framework, and they are typically and primarily used in the legal context. As in the case of other abstract concepts, institutional concepts are symbolic in nature: Their content is typically defined linguistically by a set of definitions and formal rules. At the same time, institutional objects and facts are also an outcome of intentional human activity and fulfill a specific social function. According to some theories (Searle, 1995, 2010), institutional concepts can be considered as referring to a particular kind of human-made objects that perform their function not in virtue of their physical features, as in the case of standard concrete artefacts, but via collective acceptance of the relevant rules by a given community.

The cognitive structure of institutional concepts, and their relations with artefacts, has been the object of a recent study which compared concrete and abstract standard artefacts (e.g., screwdriver vs. poetry), concrete and abstract institutional entities (e.g., signature vs. ownership), and concrete and abstract social entities (e.g., choir vs. friendship) in a property-generation task (Roversi et al., 2013). Results showed that institutional concepts are more similar to physical artefacts than to social entities (see also Noyes et al., 2018, where it is argued that the similarity between institutional and standard artefacts is close to identity in young children). Specifically, social entities such as “choir” elicited a higher proportion of contextual situations or events associated with target concepts (e.g., choir-concert), while institutional concepts such as “ownership” mainly evoked normative relations and paradigmatic examples (e.g., ownership-testament), and standard artefacts such as “screwdriver” more frequently evoke partonomic relations (e.g., screwdriver-handle). The findings also indicate that the abstract–concrete distinction is more marked within the social domain than in that of institutions and standard artefacts. The authors found that the relevance of exemplification and normative relations for institutional artefacts and the role of partonomic relations for standard artefacts do not change substantially for abstract or concrete cases, whereas the relevance of situational relations for social concepts in opposition to institutional artefacts is more specifically connected to abstract than to concrete social concepts. Even if all institutional concepts are characterized by abstract content, some of them—especially legal institutions—can be ordinary objects or states of events that acquire a new status (e.g., contract, marriage), and in this case they could be understood as artefacts in a proper sense (Burazin et al., 2018).

An important point, however, is that undoubtedly the institutional domain is rooted in some general social concepts. Concepts like “justice”, “responsibility”, “sanction”, “duty”, “rights” not only find their background in the social community but also define the general framework in which other, more technical institutional concepts like “contract”, “president”, or “marriage” are framed: While the latter are “pure institutional” concepts in the sense that they depend on formalized institutions and rules, the former could rather be qualified as “meta-institutional” concepts, because they are necessary to define the content of institutions but are not defined by those institutions. Just as there cannot be competitive games without the concept of victory, no legal system could be defined without a conception of “justice” or the concept of “duty”, “rights”, “sanctions”, and the meaning of these concepts do not depend on a specific legal system but on the general social framework (see Roversi, 2014; Lorini, 2014). In this work, while analyzing institutional concepts in opposition to other kinds of concepts, we have also added this new layer of specification: technical, “pure-institutional concepts” *vs.* more social, “meta-institutional concepts.”

We build our study about the conceptual representation of institutional concepts on three methodological assumptions.

First, to serve as a contrast for institutional concepts we have introduced both abstract and concrete categories of concepts, which are both human-related but to a different extent. We chose Theoretical concepts of mathematics and physics (e.g., sum, energy) which possess a specific object as referent, but their referent change (e.g., nuclear energy, kinetic energy) and can be ascribed to a specific area of knowledge, as in the case of Institutional ones (Villani et al., 2019). With regard to concrete concepts, we included simple and complex artefacts (e.g., hammer and computer, respectively) that have a specific function (for a review see Martin, 2007), and Food concepts (e.g., banana, pepper), that are neither artifact nor natural but that can be both depending on the circumstances (Rumiati & Foroni, 2016). We intend to verify whether only a marked distinction between concrete *vs.* abstract categories emerges or more subtle differences are present.

We also aim at verifying whether and to what extent the above-mentioned sub-groups of Pure-institutional (e.g., marriage, contract) and Meta-institutional (e.g., rights, duty) differ across the rated dimensions.

Second, in order to observe the difference among categories and to assess the role of different grounding sources, we collected ratings on 16 dimensions (see below) already used in a previous norming study (Villani et al., 2019). This aim was driven by the fact that recent proposals have indeed suggested that multiple systems—not only sensorimotor ones—are engaged in shaping conceptual representation. According to multiple representation views, abstract concepts are grounded in situational and perceptual information just like concrete concepts (Gallese & Cuccio, 2018; Pulvermüller, 2018), but they also involve to a large extent linguistic, inner (interoceptive and emotions) and social experience (Borghi et al., 2018a; Dove, 2016, 2019; Vigliocco et al., 2013; Newcombe, Campbell, Siakaluk, & Pexman, 2012; Connell, Lynott, & Banks, 2018). In this vein, we will focus on Words As social Tools proposal (WAT, Borghi & Binkofski, 2014; Borghi et al., 2018a, 2018b, 2019), according to which words can be considered as social tools useful to operate in the external environment, and as inner tools, useful to support our categorization and thought process. Since instances of abstract concepts are more heterogeneous and different from those of concrete concepts (they do not have a single referent and activate a wider variety of situations, e.g., “cause” vs. “table”), WAT proposes that linguistic mediation and social input by others are fundamental for their acquisition and that this experience influences their representation and use. In our view, linguistic and social dimensions are particularly relevant in considering Institutional concepts since they are both language-based concepts and social constructs defined collectively.

Third, we have argued that conceptual knowledge is flexibly modulated in the function of context and personal experiences (Casasanto & Lupyan, 2015). Hence, we are interested in exploring whether the conceptual representation of Institutional concepts might vary depending on the degree of the participants’ expertise in the legal framework. To this end, we contrast the ratings of each dimension obtained from experts in the legal domain, i.e., law graduates and law professionals (Law-group), with those obtained with laypeople, i.e., students and professionals of other fields (Control-group). The effect of expertise on conceptual knowledge is well-documented in the concrete domain, for example, Medin et al. (1997) have shown that taxonomists, landscape workers, and park maintenance personnel categorize concrete items such as trees differently (see also Johnson & Mervis, 1998; Tanaka & Taylor, 1991). To the best of our knowledge, only a few studies attempted to observe the impact of expertise in the abstract domain. Borghi, Caramelli and Setti (2016) obtained different definitions of three abstract concepts belonging to the “safety and security at workplace” domain from four different categories of workers. Interestingly, Roversi et al. (2013) showed that students, law graduates, and law professionals listed different features of institutional concepts: To ground the meaning of institutional concepts law professionals tended to appeal more to exemplification relations (e.g., ownership-house) than graduates in law. This testifies that graduates need to instantiate concepts in a context to represent them, while this need is not present with law professionals for whom such concepts have become familiar. Even if relevant to our aims, the results of this previous study were preliminary and pertained only to a small number of participants in each group.

Given these assumptions, we have formulated the following predictions:

1. *Institutional concepts vs. other categories*. In line with the idea that concepts are multidimensional constructs, where embodied, inner, linguistic, and social dimensions interact to a different extent (Borghi et al., 2018a, b), we expected that the ratings obtained for Institutional concepts would differ from those observed for other categories. Specifically, we hypothesize that Institutional concepts would be similar to other abstract concepts in some respects; hence they should score higher in abstractness and less in imageability than concrete ones, and they should be acquired later and more through the linguistic modality than through perception. However, because Institutional concepts serve to regulate social practices through shared sets of values, we expect them to be more linked to social dimensions when compared to theoretical concepts. In addition, we expected that they would be at least in part grounded in physical interaction, similarly to standard artefacts.
2. *Institutional concepts vary across expertise*. In keeping with research on conceptual flexibility, we predict that conceptual representation of Institutional concepts is not fixed but may be sensitive to the level of participant expertise. Specifically, we assumed that semantic representation is influenced by a complex set of experiences connected with concepts referent, including the modality of acquisition and the use of the concept in a given context. Jurists do not only have a wider and deeper knowledge of institutional concepts than laypeople, but they also acquired their meaning through formal language and by the support of competent others. Further, differently from non-experts, law-experts master the use of institutional concepts and their consequent effects (e.g., a set of acts associated with a process). Thus, we hypothesize that these acquisition experiences and the real use of institutional concepts by expertise have an influence on their representations. We predict that the representation of institutional concepts in the Law and Control groups differ in function of qualitative factors, namely the kind of experiences associated with their semantic content. Given the exploratory character of the expertise variable, we only formulate a general prediction. Since experts in law (Law-group) develop a higher level of formalism in legal framework (e.g., a precise reference to legislative institutions, doctrines) than non-experts (Control group), they might evaluate Institutional concepts as more linked to linguistic dimensions and less to sensorimotor ones. At the same time, law experts (Law-group) are typically more accustomed to the legal field than non-experts, leading to the hypothesis that they represent Institutional concepts as contextually situated, and linked to their own personal life experience, while non-experts (Control-group) might have a more abstract representation.
3. *Insight into the Institutional domain.* Given the compound structure of Institutional concepts, some differences should emerge within this category. Since Meta-institutional concepts form the conceptual background for the more specific institutional frameworks, we hypothesize that these concepts have more generic abstract features (high scores in linguistic, social, and inner dimensions), while the Pure-institutional ones bear greater similarity with “technical” and concrete concepts of artifacts (high scores in sensorimotor dimensions).

## Methods

### Participants

567 participants (409 female, *M*_age_ = 24.02; SD_age_ = 5.97) volunteered for the study. All participants were recruited among students and researchers of the University of Bologna, and people who work in the Bologna area. Participants were divided in two groups: 289 law graduates or law professionals (law-group: 204 female, *M*_age_ = 22.47; SD_age_ = 5.25; *M*_years of university education_ = 2.79; SD_years of university education_ = 1.8) and 278 graduates or professionals in fields different from law, such as philosophy, art, communication science (control-group: 205 female, *M*_age_ = 25. 64; SD_age_ = 6.24; *M*_years of university education_ = 3.71; SD_years of university education_ = 1.5). The following experiment fulfilled the ethical standard procedure recommended by the Italian Association of Psychology (AIP). All procedures were approved by the Bioethics committee of the University of Bologna. All participants gave their written informed consent to participate in the study.

### Materials

Materials consisted of 56 words. Half of them were abstract and the other half were concrete ones. For each group we considered two sub-categories of concepts: Theoretical and Institutional categories for abstract and Food and Artefacts categories for concrete concepts. A set of 14 theoretical abstract concepts (i.e., mass, acceleration, subtraction, temperature, sum, energy, liter, meter, gravitation, calculation, equation, molecule, electron, multiplication) were taken from the Villani et al. database (2019) where a cluster of physical, spatial–temporal and quantitative concepts was individuated. We included 14 Institutional concepts already used in the previous studies on Institutional concepts (Roversi et al., 2013, 2017). In selecting institutional concepts, we took care to insert half Pure-institutional (i.e., contract, state, president, marriage, parliament, trial, property) and half Meta-institutional concepts (i.e., norm, rights, duty, sanction, responsibility, validity, justice). For what concern concrete concepts, we included 14 natural Food concepts (i.e., banana, carrot, grape, strawberry, mushroom, eggplant, pepper, tomato, pumpkin, basil, apple, orange, chestnut, potato) and 14 Artefacts concepts (i.e., hammer, wheel, knife, pot, spoon, tower, umbrella, bed; screwdriver, painting; chair, sculpture, book, computer), selected from Rosa et al. (2010) and Barca et al. (2002) databases.

### Procedure

Based on the Villani et al. (2019) procedure, we asked participants to evaluate each word on 7-point Likert scale, choosing randomly only one of the following dimensions: abstractness-concreteness (ABS-CNR); imageability (IMG, Paivio, 1986); contextual availability (CA, Schwanenflugel et al., 1992); familiarity (FAM); age of acquisition (AoA) (e.g., Gilhooly & Logie, 1980); modality of acquisition (MoA, Wauters et al., 2003); social valence (SOC, Barsalou & Wiemer-Hastings, 2005); social metacognition (MESO, Borghi et al., 2019); arousal (ARO); valence (VAL) (e.g., Bradley & Lang, 1999; Warriner, Kuperman, & Brysbaert, 2013); Interoception (INT, Connell, Lynott, & Banks, 2018); metacognition (META); perceptual strength in the vision, hearing, touch, taste and smell modalities (VIS; HEA; TOU; TAS; SME, Lynott & Connell, 2013); body-object interaction (BOI; Bennett, Burnett, Siakaluk, & Pexman, 2011; Pexman, Muraki, Sidhu, Siakaluk & Yap, 2019); mouth involvement (MOUTH); hand involvement (HAND) (e.g., Borghi & Zarcone, 2016). See Table 1 for the rating instructions given to participants, and supplementary materials for theoretical discussion on each dimension.

**Table 1:** Rating instruction for each dimension

Dimension	Instruction
Abstractness-Concreteness (ABS-CNR)	rate how much each word is abstract or concrete 1 = very abstract; 7 = very concrete
Imageability (IMG)	rate how much the word arouses mental images, visual representation, a sound or some other sensory experience 1 = hardly imageable; 7 = highly imageable
Contextual availability (CA)	rate on the ease with which you can think of a context for each word 1 = very hard; 7 = very easy
Familiarity (FAM)	rate how familiar you are with the word, namely how much do you know its meaning. 1 = unfamiliar; 7 = very familiar
Age of acquisition (AoA)	indicate the age at which you think you learned each word 1 = 0–2 years; 2 = 3–4 years; 3 = 5–6 years; 4 = 7–8 years; 5 = 9–10 years; 6 = 11–12 years; 7 = 13+ years
Modality of acquisition (MoA)	rate how you think you have learned the meaning of word: through experience, through language or a combination of the two 1 = experience; 7 = language
Social valence (SOC)	rate how much the word evokes social circumstance 1 = not at all, 7 = very
Social metacognition (MESO)	rate how much you think you have or needed others to understand the meaning of each word 1 = never; 7 = almost always
Arousal (ARO)	rate how much each word evokes emotions 1 = not at all; 7 = very
Valence (VAL)	rate how much each word evokes positive or negative emotions 1 = negative emotions; 7 = positive emotions
Interoception (INT)	rate how much the word evokes an internal body state 1 = not at all; 7 = very
Metacognition (META)	rate how much the word evokes mental and cognitive processes, or more generally as it seems to you concerning processes that occur in the brain 1 = not at all; 7 = very
Perceptual strength in the vision, hearing, touch, taste, and smell modalities (VIS/HEA/TOU/TAS/SME)	rate to what extent do you experience of word through each of the five senses (i.e., “by vision”, “by touch”, “by hearing”, “by smelling” and “by tasting”) 1 = not at all; 7 = very
Body-object interaction (BOI)^a^	rate each word on the ease with which human body physically interacts with the object/entity to which the word refers 1 = easy; 7 = difficult
Mouth involvement (MOUTH)	rate how much the mouth is involved in a possible action with the named entity 1 = not at all; 7 = very
Hand involvement (HAND)	rate how much the hand is involved in a possible action with the named entity 1 = not at all; 7 = very

^a^Notice that the scale used for the body-object-interaction (BOI) is the opposite than that used in the previous literature (e.g., Siakaluk et al., 2008). Here, the low BOI score refers to things that the human body can easily interact with, (“high BOI”) and the high BOI score refers to things that are not easy for the human body to interact with (“low BOI”). The BOI results reported in Tables 2 and 3 should be interpreted according to this value

**Table 2:** Results of Generalized Estimated Equations (GEE) with *Category *(institutional, theoretical, food, artefact) as within-subject factor and *Group* (law-group and control-group) as between-subject factor

		Category				Group			Group × Category		
		Wald	*Df*	*p*		Wald	*Df*	*p*	Wald	*Df*	*p*
ABS- CNR	Institutional (*M* = 4.1) vs. Theoretical (*M* = 4) vs. Food (*M* = 6.8) vs. Artefact (*M* = 6.7)	196.417 170 211.107 207.222	3 1 1 1	**0.001** 1 **0.001** **0.001**	Law (*M* = 5.2) Control (*M* = 5.1)	0.341	1	0.599	2.088	3	0.554
IMG	Institutional (*M* = 3.9) vs. Theoretical (*M* = 3.9) vs. Food (*M* = 6.7) vs. Artefact (*M* = 6.6)	221.223 0.012 334.223 374.983	3 1 1 1	**0.001** 1 **0.001** **0.001**	Law (*M* = 5.2) Control (*M* = 4.9)	1.386	1	0.239	6.536	3	0.088
CA	Institutional (*M* = 4.9) vs. Theoretical (*M* = 4.3) vs. Food (*M* = 5.8) vs. Artefact (*M* = 5.7)	66.544 15.181 8.793 16.222	3 1 1 1	**0.001** **0.001** **0.009** **0.001**	Law (*M* = 5.0) Control (*M* = 5.4)	2.079	1	0.149	28.854	3	**0.001**
FAM	Institutional (*M* = 5.9) vs. Theoretical (*M* = 5.0) vs. Food (*M* = 6.3) vs. Artefact (*M* = 6.1)	40.988 31.405 3.315 1.413	3 1 1 1	**0.001** **0.001** 0.206 0.704	Law (*M* = 5.6) Control (*M* = 6.1)	2.452	1	0.117	40.513	3	**0.001**
AoA	Institutional (*M* = 4.6) vs. Theoretical (*M* = 4.3) vs. Food (*M* = 2.1) vs. Artefact (*M* = 2.4)	352.688 10.654 353.021 397.889	3 1 1 1	**0.001** **0.003** **0.001** **0.001**	Law (*M* = 3.0) Control (*M* = 3.3)	1.462	1	0.227	16.806	3	**0.001**
MoA	Institutional (*M* = 5.1) vs. Theoretical (*M* = 5) vs. Food (*M* = 1.9) vs. Artefact (*M* = 2.3)	93.817 2.033 209.358 209.422	3 1 1 1	**0.001** 0.462 **0.001** **0.001**	Law (*M* = 3.2) Control (*M* = 3.3)	0.269	1	0.604	1.085	3	0.781
SOC	Institutional (*M* = 5.7) vs. Theoretical (*M* = 2.8) vs. Food (*M* = 2.2) vs. Artefact (*M* = 3)	81.594 132.286 125.665 114.944	3 1 1 1	**0.001** **0.001** **0.001** **0.001**	Law (*M* = 3.0) Control (*M* = 3.4)	0.685	1	0.408	3.911	3	0.271
MESO	Institutional (*M* = 3.2) vs. Theoretical (*M* = 3.2) vs. Food (*M* = 1.2) vs. Artefact (*M* = 1.4)	284.447 0.246 75.445 79.852	3 1 1 1	**0.001** 1 **0.001** **0.001**	Law (*M* = 2.1) Control (*M* = 1.9)	0.433	1	0.511	6.564	3	0.087
ARO	Institutional (*M* = 4) vs. Theoretical (*M* = 2.7) vs. Food (*M* = 2.7) vs. Artefact (*M* = 2.9)	68.102 77.634 31.511 49.081	3 1 1 1	**0.001** **0.001** **0.001** **0.001**	Law (*M* = 2.8) Control (*M* = 3.1)	0.709	1	0.400	6.428	3	0.093
VAL	Institutional (*M* = 4.6) vs. Theoretical (*M* = 4.1) vs. Food (*M* = 5) vs. Artefact (*M* = 4.5)	28.779 6.336 7.142 0.188	3 1 1 1	**0.001** **0.035** **0.023** 1	Law (*M* = 4.7) Control (*M* = 4.3)	3.045	1	0.081	12.337	3	**0.006**
INT	Institutional (*M* = 4) vs. Theoretical (*M* = 3.1) vs. Food (*M* = 3.2) vs. Artefact (*M* = 3.1)	24.954 14.664 7.195 19.773	3 1 1 1	**0.001** **0.001** **0.022** **0.001**	Law (*M* = 3.4) Control (*M* = 3.3)	0.036	1	0.849	0.431	3	0.934
META	Institutional (*M* = 4.7) vs. Theoretical (*M* = 3.6) vs. Food (*M* = 2.7) vs. Artefact (*M* = 3.3)	64.688 33.221 51.286 43.077	3 1 1 1	**0.001** **0.001** **0.001** **0.001**	Law (*M* = 3.3) Control (*M* = 3.8)	3.002	1	0.083	6.088	3	0.107
VIS	Institutional (*M* = 4.4) vs. Theoretical (*M* = 4.5) vs. Food (*M* = 5.9) vs. Artefact (*M* = 6.0)	56.441 0.165 36.533 51.110	3 1 1 1	**0.001** 1 **0.001** **0.001**	Law (*M* = 5.3) Control (*M* = 4.9)	1.329	1	0.249	7.583	3	0.055
HEA	Institutional (*M* = 3.5) vs. Theoretical (*M* = 2.5) vs. Food (*M* = 1.7) vs. Artefact (*M* = 3)	94.733 39.864 76.163 6.930	3 1 1 1	**0.001** **0.001** **0.001** **0.025**	Law (*M* = 2.7) Control (*M* = 2.4)	0.702	1	0.402	6.096	3	0.107
TOU	Institutional (*M* = 2) vs. Theoretical (*M* = 2.6) vs. Food (*M* = 4.9) vs. Artefact (*M* = 4.8)	269.925 30.625 136.141 336.637	3 1 1 1	**0.001** **0.001** **0.001** **0.001**	Law (*M* = 3.6) Control (*M* = 3.0)	3.654	1	0.056	26.073	3	**0.001**
TAS	Institutional (*M* = 1.3) vs. Theoretical (*M* = 1.4) vs. Food (*M* = 6) vs. Artefact (*M* = 1.6)	1099.220 0.991 661.493 18.369	3 1 1 1	**0.001** 0.959 **0.001** **0.001**	Law (*M* = 2.0) Control (*M* = 1.9)	0.587	1	0.443	1.642	3	0.650
SME	Institutional (*M* = 1.5) vs. Theoretical (*M* = 1.6) vs. Food (*M* = 4.8) vs. Artefact (*M* = 2.2)	251.863 1.088 201.666 76.979	3 1 1 1	**0.001** 0.891 **0.001** **0.001**	Law (*M* = 2.4) Control (*M* = 2.0)	2.249	1	0.134	2.040	3	0.564
BOI	Institutional (*M* = 4.1) vs. Theoretical (*M* = 3.6) vs. Food (*M* = 1.7) vs. Artefact (*M* = 2)	42.130 6.441 52.684 50.701	3 1 1 1	**0.001** **0.033** **0.001** **0.001**	Law (*M* = 2.5) Control (*M* = 2.8)	0.968	1	0.325	0.639	3	0.887
MOUTH	Institutional (*M* = 3.9) vs. Theoretical (*M* = 2.8) vs. Food (*M* = 5.1) vs. Artefact (*M* = 2.8)	43.359 21.312 7.802 14.301	3 1 1 1	**0.001** **0.001** **0.016** **0.001**	Law (*M* = 3.7) Control (*M* = 3.3)	2.344	1	0.126	1.021	3	0.796
HAND	Institutional (*M* = 2.9) vs. Theoretical (*M* = 2.9) vs. Food (*M* = 4.5) vs. Artefact (*M* = 4.8)	66.526 0.025 18.172 45.339	3 1 1 1	**0.001** 1 **0.001** **0.001**	Law (*M* = 3.6) Control (*M* = 3.8)	0.456	1	0.500	5.393	3	0.145

Planned single contrast for the *Category*, between the scores obtained for institutional concepts vs. food, artefacts, and theoretical concepts, are reported. In bold are reported significant results

**Table 3:** Results of Generalized Linear Models (GLM) applied for each dimension with the *Type of Institutional* (pure-institutional and meta-institutional) as within-subject factor and *Group* (law-group and control-group) as between-subject factor.

	Group			Type of institutional			Group × Type		
	Wald	*Df*	*p*	Wald	*Df*	p	Wald	*Df*	*p*
ABS-CNR	0.612	1	0.434	29.960	1	**0.001**	3.888	1	**0.049**
	Law (*M* = 4.1) Control (*M* = 3.7)			Pure (*M* = 4.6) Meta (*M* = 3.3)					
IMG	3.073	1	0.080	45.000	1	**0.001**	3.683	1	0.055
	Law (*M* = 4.1) Control (*M* = 3.4)			Pure (*M* = 4.9) Meta (*M* = 2.8)					
CA	4.789	1	**0.029**	24.696	1	**0.001**	7.607	1	**0.006**
	Law (*M* = 5.6) Control (*M* = 4.6)			Pure (*M* = 5.6) Meta (*M* = 4.6)					
FAM	4.648 Law (*M* = 6.3) Control (*M* = 5.6)	1	**0.031**	5.791 Pure (*M* = 5.8) Meta (*M* = 6.0)	1	**0.016**	0.376	1	0.540
AoA	12.269	1	**0.001**	2.041	1	0.153	0.555	1	0.456
	Law (*M* = 4.0) Control (*M* = 5.0)			Pure (*M* = 4.4) Meta (*M* = 4.6)					
MoA	9.924	1	**0.002**	0.001	1	0.973	5.643	1	**0.018**
	Law (*M* = 5.0) Control (*M* = 5.7)			Pure (*M* = 5.3) Meta (*M* = 5.3)					
SOC	1.837	1	0.175	0.084	1	0.772	0.084	1	0.772
	Law (*M* = 6.1) Control (*M* = 5.5)			Pure (*M* = 5.8) Meta (*M* = 5.8)					
MESO	0.024	1	0.878	10.057	1	**0.002**	0.052	1	0.819
	Law (*M* = 3.2) Control (*M* = 3.1)			Pure (*M* = 2.9) Meta (*M* = 3.4)					
ARO	1.222	1	0.269	18.606	1	**0.001**	0.073	1	0.786
	Law (*M* = 4.3) Control (*M* = 3.8)			Pure (*M* = 3.5) Meta (*M* = 4.7)					
VAL	14.994	1	**0.001**	9.006	1	**0.003**	5.894	1	**0.015**
	Law (*M* = 5.2) Control (*M* = 4.1)			Pure (*M* = 4.4) Meta (*M* = 5.0)					
INT	0.008	1	0.929	21.134	1	**0.001**	0.454	1	0.500
	Law (*M* = 3.8) Control (*M* = 3.8)			Pure (*M* = 3.4) Meta (*M* = 4.2)					
META	0.226	1	0.635	10.515	1	**0.001**	0.769	1	0.381
	Law (*M* = 4.8) Control (*M* = 4.6)			Pure (*M* = 4.3) Meta (*M* = 5.1)					
VIS	4.129	1	**0.042**	26.986	1	**0.001**	0.000	1	0.996
	Law (*M* = 5.0) Control (*M* = 3.9)			Pure (*M* = 5.2) Meta (*M* = 3.7)					
HEA	5.589	1	**0.018**	0.000	1	1.000	0.000	1	1.000
	Law (*M* = 4.1) Control (*M* = 2.7)			Pure (*M* = 3.3) Meta (*M* = 3.3)					
TOU	3.439	1	0.064	0.436	1	0.509	0.011	1	0.916
	Law (*M* = 2.1) Control (*M* = 1.4)			Pure (*M* = 1.8) Meta (*M* = 1.7)					
TAS	3.874	1	**0.049**	1.103	1	0.294	1.103	1	0.294
	Law (*M* = 1.1) Control (*M* = 1.0)			Pure (*M* = 1.0) Meta (*M* = 1.0)					
SME	1.611	1	0.204	0.057	1	0.812	0.057	1	0.812
	Law (*M* = 1.5) Control (*M* = 1.2)			Pure (*M* = 1.3) Meta (*M* = 1.3)					
BOI	3.584	1	0.058	1.670	1	0.196	1.123	1	0.289
	Law (*M* = 3.5) Control (*M* = 4.5)			Pure (*M* = 3.8) Meta (*M* = 4.1)					
MOUTH	2.072	1	0.150	6.995	1	**0.008**	1.508	1	0.219
	Law (*M* = 4.3) Control (*M* = 3.5)			Pure (*M* = 4.0) Meta (*M* = 3.7)					
HAND	0.793	1	0.373	3.544	1	0.060	1.690	1	0.194
	Law (*M* = 2.4) Control (*M* = 2.9)			Pure (*M* = 2.8) Meta (*M* = 2.5)					

In bold are reported significant results

### Data collection

For each dimension, a survey was created and administered through Google Form. Following the consent form and instruction page, participants were presented the full list of stimuli in randomized order. Notice that each rating was administered in order to obtain an equal sample of participants into the Law and Control groups, resulting in at least 15 participants per group in each dimension (ABS-CNR = 20, 22; IMG = 18, 15; CA = 19,16; FAM = 15, 15; AoA = 21, 21; MoA = 16, 16; SOC = 16,15; MESO = 18,18; ARO = 17, 17; VAL = 18,18; INT = 15,15; META = 23, 17; VIS, HEA, TOU, TAS, SME = 16, 16; BOI = 23, 23; MOUTH = 15, 18; HAND = 19, 16; Law and Control participants, respectively for each dimension).

### Statistical analysis

The analysis was conducted on the rating values obtained in each dimension. Generalized Estimated Equations (GEE) with Gamma function^1^ was used instead of standard analysis of variance since rating values were discrete rather than continuous variables (Dixon, 2008).

For each dimension, the factors taken into consideration were *Category* (institutional, theoretical, food and artefact) as within-subject factor and *Group* (Law-group and Control-group) as between-subject factor. Since we were particularly interested to verify whether institutional concepts differed from other categories of concepts, in each dimension, we performed a planned single contrast between the rating values obtained for Institutional concepts vs. Food, Artefacts and Theoretical concepts.

To explore the subtle differences within Institutional concepts, we performed additional Generalized Linear Models (GLM) with Gamma function,^2^ considering *Type of Institutional* (Pure-institutional and Meta-institutional) as within-subject factor and  *Group* (Law-group and Control-group) as between-subject factor.

## Results

**Fig. 1:**
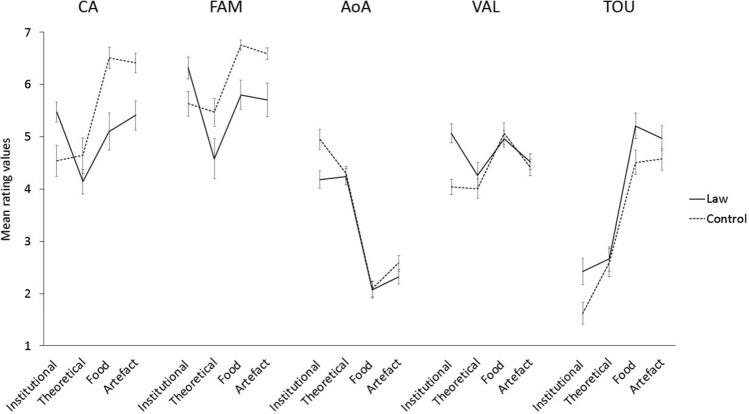
Mean rating values for *Group* (law-group and control-group) as a function of the *Category* (institutional, theoretical, food, artefact) in the following dimension: contextual availability (CA), familiarity (FAM), age of acquisition (AoA), valence (VAL), perceptual strength in touch (TOU). Error bars indicate standard errors of the mean

Results of the GEE models are reported in detail in Table 2 for each dimension. They showed a significant main effect *Category* in all rated dimensions, demonstrating that the categories (i.e., institutional, theoretical, food and artefact) are widely different from each other (see planned contrast in Table 2 for a comparison across categories). In all dimensions, the main effect *Group *was not significant, revealing that, overall, no difference emerged between the Law-group and the Control-group. Interestingly, however, the interaction between *Category* and *Group* was significant in some dimensions (i.e., CA, FAM, AoA, VAL, TOU see Fig. 1). Means, for each dimension, in the function of the interaction *Group x Category* are reported in the supplementary materials.

**Fig. 2:**
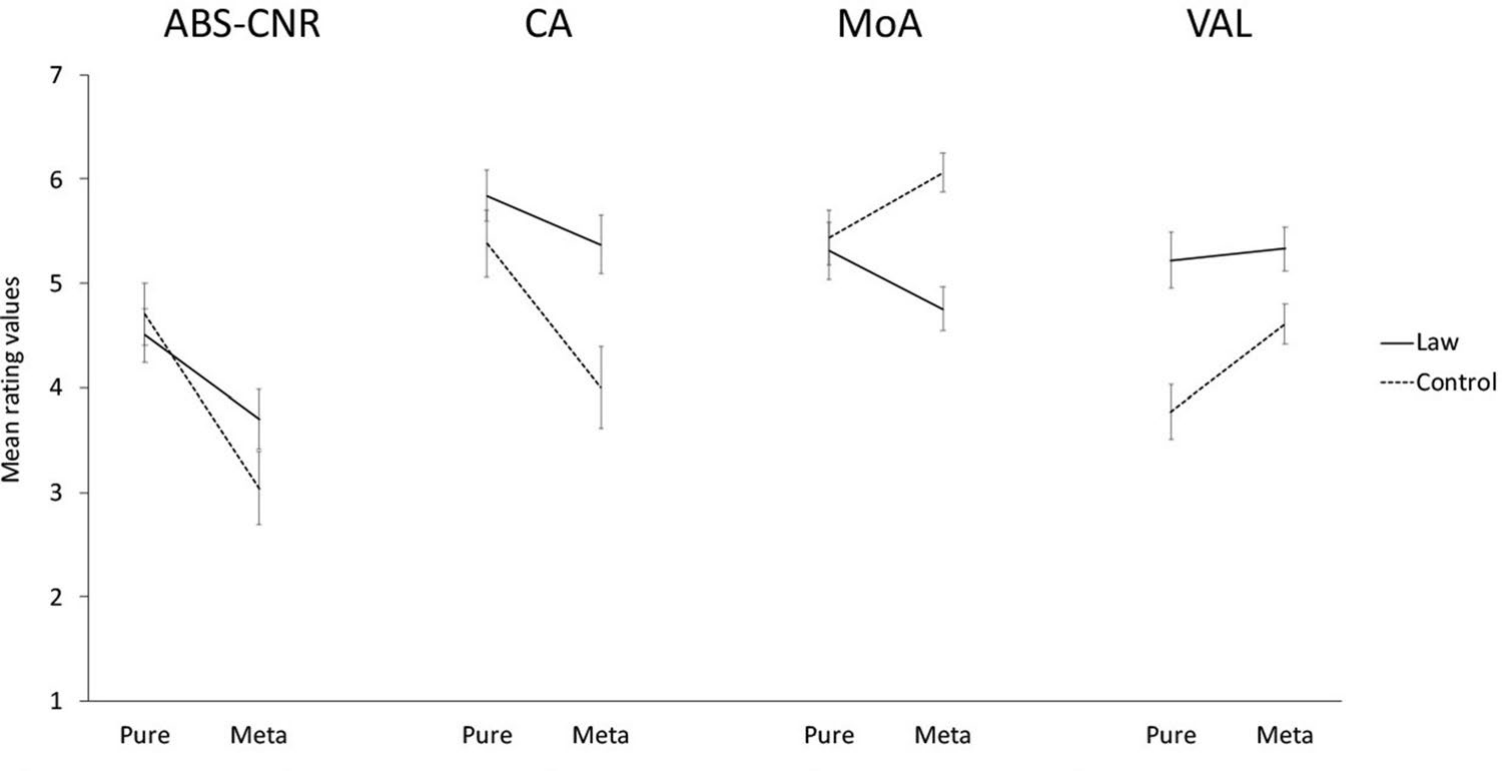
Mean rating values for *Group* (law-group and control-group) as a function of *Type of Institutional *(pure, meta) in the following dimension: abstractness-concreteness (ABS-CNR), contextual availability (CA), modality of acquisition (MoA), valence (VAL). Error bars indicate standard errors of the mean

Results of the GLM are reported in detail in Table 3. They revealed that the main effect *Type of Institutional* was significant in some dimensions (i.e., ABS-CNR, IMG, CA, MESO, ARO, VAL, INT, META, VIS, MOUTH), thus the two type of institutional concepts (i.e., Pure and Meta) significantly differ across specific dimensions. The main effect *Group* was significant in few dimensions (i.e., CA, FAM, AoA, MoA, VAL, VIS, HEA, TAS), revealing that, overall, a difference emerged between the Law-group and the Control-group. Crucially, the interaction *Group x Type of Institutional *was significant in some dimensions (i.e., ABS-CNR, CA, MoA, VAL; see Fig. 2) Means, for each dimension, in function of the interaction *Group x Type of Institutional *are reported in the supplementary materials.

## Discussion

Overall, our findings confirm that the categories we investigated (institutional, theoretical, food, and artefact) are widely different from each other and shed light on the more subtle differences among a specific domain of abstract concepts and other categories of concepts. Crucially, some of the obtained differences were affected by individual experience. Here, we will summarize and discuss firstly the results on Institutional concepts comparing them with concrete concepts and all other categories, secondly Institutional concepts related to group differences and thirdly, subtypes of Institutional concepts.

### Institutional concepts vs. other categories

From our results, we could make a distinction between (1) the dimensions in which Institutional concepts differ only from concrete concepts, (2) the dimensions in which they differ from all categories, including Theoretical concepts, and (3) the dimension in which Institutional concepts differ only from Theoretical concepts but not from all other categories.

1. The first case regards the dimensions of abstractness-concreteness (ABS-CNR), imageability (IMG), modality of acquisition (MoA), social metacognition (MESO), vision, taste and smell modalities (VIS; TAS and SME) and hand involvement (HAND). Since in these dimensions Institutional concepts differ only from both kinds of concrete concepts (i.e., food and artifact), this means that Institutional and Theoretical concepts do not differ, but they share some properties that make them dissimilar to concrete concepts. In fact, compared to concrete concepts they are both evaluated as highly abstract and less imageable; they are mainly linguistically acquired (high MoA) and they involve a stronger need to rely on others, in order to understand their meaning (high MESO). In addition, abstract concepts are less experienced through perceptual modalities compared to concrete ones (i.e., vision, taste, and smell), and the hand effector is less involved in a possible action with them.
  Overall, results confirm that abstract concepts are more detached from perceptual modalities (Barsalou, 2003). Crucially, they also show other components which contribute to the grounding of abstract concepts, in line with multiple representation views. The high values of the modality of acquisition and social metacognition for abstract concepts fully confirm the prediction of the WAT proposal (Borghi et al., 2018a, 2019), and testifies that the higher the abstractness of words, the more we need others’ support, and that linguistic and social experiences play a crucial role in representing abstract concepts.
2. The second case concerns dimensions of context availability (CA), age of acquisition (AoA), social valence (SOCIAL), arousal (ARO), valence dimension (VAL), interoception (INT), metacognition (META), hearing (HEA), touch (TOU) modalities, body-object interaction (BOI) and mouth involvement (MOUTH). These results suggest that Institutional concepts possess specific features that distinguish them from other concepts, including abstract ones. Compared to Theoretical, Food and Artefact concepts, Institutional concepts are more linked to a specific context (high CA); they are acquired later (high AoA); they evoke more social experience (high SOC); they are characterized by evoking inner emotional and cognitive states (i.e., high ARO, INT, META); they denote entities with which is not easy to physically interact (high BOI, that correspond to low BOI score according to the scale used in previous studies, e.g., Siakaluk et al., 2008); they require a higher mouth and hearing activation (high HEA and MOUTH) but less tactile interaction (low TOU). Institutional concepts were perceived as more positive than Theoretical concepts, and more negative than Food concepts (higher and lower values in the VAL, respectively), however, they did not differ from Artefacts.
  From an embodied perspective, it is interesting to note that Institutional concepts were characterized by both mouth and hearing dimensions, suggesting a clear link to the use of language in a social context. This pattern is in keeping with the specific claim of WAT that abstract conceptual representation not only lead to the activation of linguistic and social experiences but also engages the mouth motor systems to a larger extent than concrete concepts. Three different mechanisms, that are not necessarily exclusive, might underlie such a mouth activation (for details see Borghi et al., 2019): (A) Re-enactment of the past experience of acquiring abstract concepts, which typically occurred through the verbal linguistic mediation (Granito et al., 2015; Barca et al., 2017, 2020; Mazzuca et al., 2018). (B) Re-explanation to ourselves of the word meaning, through the use of inner speech (Zannino et al., 2021; Villani et al., 2021; Dove et al., 2020; Borghi, 2020). (C) A motor preparation to ask additional explanations of word meaning to others, derived from the feeling of uncertainty and the metacognitive awareness that the owned knowledge of that concept is scarce and not adequate (i.e., Social Metacognition, Borghi et al., 2019; Fini & Borghi, 2019; see Borghi, Fini, & Tummolini, 2021; Borghi, 2020; Dove et al., 2020; see also Prinz, 2012; Shea, 2018). The case of Institutional concepts, which can be considered as highly complex words, may include either the mediation of inner and overt speech or simulation of listening to someone else speaking. Collecting new evidence will be pivotal to disentangle between these mechanisms with respect to specific sub-kind of concepts.
3. The third case concerns familiarity dimensions (FAM). Planned contrasts showed that Institutional concepts were rated as more familiar than Theoretical concepts, while no difference emerged with Food and Artefact concepts. This means that the participants are more familiar with meanings conveyed by Institutional concepts compared to other concepts with the same abstractness level, namely physical and mathematical terms.

Generally, our main prediction was confirmed by results: social/linguistic experiences contribute to the shaping of Institutional concepts. However, the social context and hence the inter-subjective dimension resonates with a distinctively intra-subjective dimension in the conceptualization of institutions, which are perceived as being dependent on our mental states and involve a considerable state of emotional arousal. Apart from the quantity of arousal, which is significant, also the quality of the arousal behind institutional concepts is particular interesting: when compared with abstract theoretical concepts, Institutions require some degree of positive emotional support, but on the whole, the weak positive valence that seems also typical of tools and other artifacts, not the strong positive valence that is elicited by basic needs. This emotional element will be further specified below in the light of the distinction between Pure-institutional and Meta-institutional concepts.

### Institutional concepts vary across expertise

In line with our prediction, we found that the conceptualization of Institutional concepts is modulated by different kinds of personal experience. Results (see Fig. 1) showed that participants who have more experience in the legal framework (i.e., law group), compared to those that haven’t (i.e., control group), rated Institutional concepts as more contextually situated (high CA), slightly more familiar (FAM), as acquired earlier (low AoA), and as associated to a more positive valence (high VAL). Furthermore, compared to participants in the Control-group, those in the Law-group engage in more tactile experience during Institutional concepts processing (high TOU). The present dimensions are often used as indexes of concreteness/abstractness of a word. Typically, abstract words are less associated with a single context (Schwanenflugel et al., 1992), are acquired later than concrete ones (Gilhooly & Logie, 1980; Carroll & White, 1973), even if, according to some authors, affective experience may provide a bootstrapping mechanism for acquisition of abstract words (Vigliocco et al., 2013). Finally, recent studies, which obtained norms on 400 words on perceptual strength of each of the five senses, have shown that not only sight but also touch plays a critical role in considering a word as concrete (Connell & Lynott, 2012; Lynott & Connell, 2013). It is noteworthy that Law-group results present an opposite pattern to those commonly observed in literature. This suggests that people who have greater experience in the legal field tend to perceive Institutional concepts as more embodied and concrete even if they developed complex knowledge of these categories. Our results partially support the idea of an artifactual nature of institutional concepts. Only law experts, who are aware of all nuances of the meaning of institutional concepts, considered them as concrete objects that possess useful functions, as testified by the higher scores in tactile modality and positive valence. Finally, it does not surprise that Institutional concepts were evaluated as slightly more familiar by Law-group (*M* = 6.3) than Control-group (*M* = 5.6), since jurists have more knowledge of the concepts about which they are experts. Importantly, results observed in the other dimensions suggest that the law experts and non-experts do not differ only for the *quantity* of knowledge associated with institutional concepts, but rather bear a *qualitative* difference in the content of semantic representations.

### Insight into the Institutional domain: Pure vs. Meta-institutional concepts

Crucially, our findings also support a fine distinction between Pure and Meta-institutional concepts. Pure-institutional concepts obtained high scores in concreteness (ABS-CNR), imageability (IMG), context availability (CA), visual modality dimensions (VIS) and high mouth effector activation (MOUTH). Meta-institutional, compared to Pure-institutional concepts, are characterized by their being more familiar (FAM), by their relying to a larger extent on the competence of others (high MESO), and by their activating emotions (high ARO), mainly positively connoted emotions (high VAL), inner states and process (high INT; high META). Overall, while the Pure-institutional elicited a higher proportion of exteroceptive experiences, the Meta-institutional mainly rely on inner and metacognitive experiences.

At first glance, the absence of the association between the mouth and social metacognition dimensions conflicts with WAT’s proposal regarding an increase of mouth activation for processing of most abstract concepts. However, the mouth motor system can be also activated by the semantic content of the words. A good example is provided by food concepts and face-related action words (e.g., talk), the content of which directly refers to mouth actions (for neuroimaging evidence see Dreyer & Pulvermüller, 2018). Likewise, although Pure-institutional concepts were linked to concrete components, a mouth involvement emerged. Specifically, Pure-Institutional concepts included words like “parliament”, “president” or “process” that refer to entities and social practices that are inevitably based on the use of language, hence the mouth motor system. Meta-Institutional concepts, instead, include words like “duty”, “responsibility” or “justice” whose content varies dynamically depending on the context. One could speculate that mouth engagement is of paramount importance for Institutional concepts, and especially for Pure ones, since they are acquired through other words (both verbal or written modality), most often by explanations by others in a formal context (e.g., at the university), and because their content refers to situations in which language/dialogue is used. Furthermore, our results showed that Meta-institutional concepts are vaguer and more difficult to interpret than Pure, technical Institutional concepts, but also that they are more familiar and generally bear the weight of the emotional adhesion behind our institutional framework. This seems to strongly support the intuition, mentioned at the beginning, of a distinction between Pure-institutional concepts perceived as technical tools to achieve normative effects and Meta-institutional concepts forming the socially supported background (a broad and vague background, in need of constant reinterpretation) within which those tools are inscribed.

Regardless of the type, we found that expertise has an influence on the representation of institutional concepts. Overall, the Law-group was more familiar with both Pure and Meta institutional concepts than the Control group (FAM). Compared to Control-group, the Law-group also rated both types of institutional concepts as acquired earlier in their childhood (AoA) and considered those concepts as more related to hearing (HEA), vision (VIS), and taste modality (TAS). These differences confirm that the law experts are more acquainted with legal concepts than non-experts, but also suggest that their higher sensitivity to Institutional concepts is reflected in a higher involvement of sensorimotor experiences.

Importantly, results (see Fig. 2) also showed that the two types of institutional concepts were differently evaluated by the two groups. In the ABS-CNR dimensions, Pure-institutional concepts were rated as more concrete than Meta-institutional concepts by both groups. However, Meta-institutional were rated more concrete by the Law-Group than the Control-group, indicating that for law experts Meta-institutional concepts also possess concrete aspects. Likewise, in CA dimensions the Law-group considered both concepts as highly contextually situated compared to the Control-group. Interestingly, the difference between the groups emerged to great extent for Meta-institutional concepts: Law-group evaluated them as easier to associate to a context compared to the Control-group. In the MoA dimension, for the Pure-institutional concepts, the modality of the acquisition was the same for the two groups, while for the Meta-institutional concepts, in the Law group, compared to Control-group, linguistic acquisition was less involved. Finally, the effect of expertise clearly emerged in the valence dimension (i.e., VAL): overall, the Law-group has associated more positive scores to both kinds of institutional concepts compared to the Control-group. This result is in line with evidence showing that higher processing fluency is associated with a feeling of pleasantness (e.g., Topolinski & Strack, 2009). Specifically, an equally positive valence to Pure and Meta-institutional concepts were given by the Law-group, while the Control-group associated more negative emotions to Pure-institutional concepts than Meta-institutional ones. These results show that expertise modulates both the distinction between Pure-institutional and Meta-institutional concepts and the vagueness of the latter. In general, experts in law find it less difficult to contextualize general concepts like justice, duty, rights, than non-experts, and in this sense, for them these concepts are already “embedded” in specific institutional frameworks. Moreover, experts in law show emotional support not only to general social ideas connected with institutions but also to specific institutional frameworks.

### Expertise and variability

We argued that all concepts have fuzzy boundaries, and a simple concrete-abstract dichotomy is not sufficient to account for the entire semantic variability within either domain, since many abstract concepts have concrete components and vice versa (Barsalou et al., 2018; Wiemer‐Hastings & Xu, 2005).

Our findings contribute to the literature on conceptual representations, highlighting the importance of individual variability. We found interesting differences in conceptualizing institutional concepts depending on the level of expertise of participants, in line with research on conceptual flexibility (Barsalou, 1993, 1987). Importantly, the effect of expertise is not limited to how familiar the participants are with the word, but rather to different kinds of content tied by their experience with concept referent. The law experts tend to consider Institutional concepts as less abstract and more linked to sensorimotor experiences than non-experts; and represented both Pure and Meta institutional concepts as equally contextually situated and grounded them in emotional (positive) states. Our results suggest that the higher the level of competence possessed, the higher the degree of direct ‘embodied’ experience re-enacted by a given concept. Similar findings were shown in the concrete domains. For example, Hoenig et al. (2011) using fMRI showed that only professional musicians activate the auditory association cortex when identifying pictures of musical instruments but not with pictures of another object. With regards to abstract domains, Mazzuca et al. (2020) have recently shown with a property generation task that people who differ in gender identity, sexual orientation, and gender-normativity stressed different aspects of concept of gender. Specifically, normative individuals mainly relied on a bigenderist conception (e.g., male/female), while non-normative individuals produced more properties related to social context (e.g., queer, fluidity, construction).

Further research should deeply explore whether the correlation between expertise and high grounding representation is more prominent for some concepts, for example, the more difficult and technical like “entropy” or whether it is also extended to the social-emotional connotated ones, like “ethnicity”, and whether the same effects emerge using language production tasks in which an easy and precise activation of appropriate word-associations might be observed at the increasing of expertise.

### Implications for conceptual jurisprudence

These results could have a significant import for legal theory and the theory of social institutions more in general. The ultimate dependence of institutional structures on mental states is here taken as a premise, and it justifies the methodological assumption that an analysis of conceptual content can provide us with new insights to deal with questions about the nature of law and of social institutions. Even though we considered only a few representative stimuli for each subtype of institutional concepts, the concepts chosen for the institutional domain have not been selected arbitrarily: they capture several aspects of that domain, thus providing us with a picture that, though partial, nevertheless is quite comprehensive. Not only do Pure-institutional concepts denote paradigms of institutions both in private and public law, but they are also quite diverse, denoting legal roles (i.e., “President”), institutions (i.e., “State”), transactions (i.e., “contract”), procedures (i.e., “trial”). Also, the selection of Meta-institutional includes entities (i.e., “rules”), modalities (i.e., “duty”, “rights”), values (i.e., “justice”), statuses (i.e., “responsibility”, “validity”). The most important legal-theoretical consideration coming from these results is that the idea of institutions being supported by general acceptance must be significantly shaded. First of all, even though institutional concepts have a stronger dependence on linguistic and social factors than other kinds of abstract concepts, as one would expect (institutions are activated by way of declarations and taught by way of definitions and rules), they also elicit a high degree of emotional arousal: hence, social acceptance is not simply a matter of cold “cognitive” states and beliefs—a picture that, for example, theories of collective intentionality (Searle, 2010; Tuomela, 2013; Gilbert, 2014) convey—but of personal emotional involvement. Second, the way in which institutional concepts are experienced varies on the basis of the kind of institutional concept and of the subject who is internalizing it. The distinction between Pure-institutional concepts, referring to actual institutional entities or practices that have normative effects in a social context (marriage, contract, president, etc.), and Meta-institutional concepts referring to the general ideas and values that provide the conceptual background for law and social regulation (justice, validity, responsibility, sanction), finds a strong support in this work, because these two sub-groups of Institutional concepts show significant distinctions in most dimensions. Previous work on the relation between institutions and artifacts (Burazin et al., 2018) is also confirmed but, interestingly, only in connection with expertise: While, in general, institutional concepts are perceived as abstract, linguistic, and difficult to imagine, jurists, in particular, conceive institutions as more context-related, “physical”, technical tools that can be “touched”. Apart from this technical part of the institutional domain, Meta-institutional concepts bear the most part of the emotional weight of institutions: Here, too, expertise becomes relevant, because jurists tend to connect positive values to institutional frameworks—they are not simply emotionally aroused, they rather embrace the institutional context—and tend to blur the emotional dichotomy between general concepts and technical institutions, conceiving these two aspects as connected and therefore shaping general ideas in the form of specific regulations of behavior. All of this can be interpreted as indirect support to the legal-theoretical tenet that, though legal institutions require general conformity in a community, legal officials play a peculiar role in supporting them and in building the overall institutional structure (Hart, 1994).

### Conclusions

In the current literature, while sub-categories of concrete concepts have been identified, less is known about the distinctions within the abstract domain. In this study, we provide a fine-grained characterization of Institutional concepts, investigating their similarity and difference with respect to other kinds of concepts on several psycholinguistic and semantic dimensions. Overall, this study shows the peculiarity of Institutional concepts that, within the abstract domain, can be considered as a particular kind in which emotional, social, and physical aspects coexist. These components might vary dynamically as a function of personal life experiences and expertise. Importantly, we found that the higher the expertise level, the stronger are the concrete determinants. Future research on different kinds of abstract concepts should shed light on whether such a link between concrete and embodied determinants of abstract concepts and a higher level of expertise holds across domains.

## Supplementary Information

Below is the link to the electronic supplementary material.

Supplementary file1 (DOCX 16 KB)

Supplementary file2 (DOCX 24 KB)

## Footnotes

1 GEE model with Linear function was also conducted. Results did not differ from those obtained using Gamma function.

2 GLM model with Linear function was also conducted. Results did not differ from those obtained using Gamma function.
