# Supplementary material for: Is justice grounded? How expertise shapes conceptual representation of institutional concepts
Source: Psychol Res. 2021 Mar 7;86(8):2434–50. doi: 10.1007/s00426-021-01492-8 (PMC9674748; doi:10.1007/s00426-021-01492-8)
Supplement: Supplementary file 1 — Supplementary file1 (DOCX 16 KB) [file 426_2021_1492_MOESM1_ESM.docx]

**Supplementary materials 1**

**Rating dimensions**

In this study, we tested whether individuals’ expertise leads to a different rating of specific kinds of abstract (i.e., Institutional and Theoretical) and concrete concepts (i.e., Food and Artefacts) along a series of dimensions. Building on a previous norming study (Villani et al., 2019), we introduced not only the classical dimensions, typically used to identify abstract and concrete concepts, but also some novel dimensions selected on the basis of the theoretical predictions of the major current views on abstract concepts. Differently from Villani et al. (2019), here concreteness and abstractness ratings were provided in a single scale (e.g., Paivio, 1986; Barca, Burani, & Arduino, 2002) and Familiarity (e.g., Gilhooly & Logie, 1980) and Valence dimension was introduced (e.g., Bradley & Lang, 1999; Warriner, Kuperman, & Brysbaert, 2013). We detailed all dimensions below, together with their theoretical reasons.

Classical dimension
We first included dimensions that, according to different theories, are considered crucial to distinguish concrete and abstract words, i.e., abstractness/concreteness (ABS-CNR), Imageability (IMG; Paivio, 1986), Context Availability (CA; Schwanenflugel et al., 1992) and Familiarity (FAM). Ratings on Imageability and Context Availability are related to the two classical theories of abstract concepts: the Dual-Coding Theory (DCT; Paivio, 1990) and the Context Availability Theory (Schwanenflugel et al., 1992). According to DCT, the concreteness effect, i.e., the fact that concrete concepts are processed and recalled better than abstract concepts, is due to their higher imageability, while, according to Context Availability theory, it is due to the reduced number of associated contexts. However, recent studies have shown that imageability is correlated with concreteness but cannot be treated as equivalent (Kousta et al., 2011); and that perceptual strength explains concreteness effect better than imageability (Connell & Lynott 2012).

Novel dimensions
The dimensions we introduced were aimed to test claims deriving from the recent Multiple Representation View (for a review see Borghi et al., 2017). These views contend that in order to fully account for abstract concept representation, likely linguistic, social, and inner experiences are involved beyond sensorimotor systems. Therefore, we considered the following dimensions related to the just-mentioned experiences.

*Language.*
Literature on words acquisition has demonstrated that two dimensions are strongly correlated to concreteness level: Age of Acquisition (AoA, Gilhooly & Logie, 1980) and Modality of Acquisition (MoA; Wauters et al., 2003). When compared to concrete words, abstract words are typically acquired later (AoA), and more through language rather than through perceptual modalities (MoA) (Villani et al., 2019).

*Sociality.*
To explore the role of social dimension for abstract and concrete concepts, we considered social valence (SOC; i.e., how much a concept evokes social situations) and social metacognition (MESO; i.e., how much we need to consult other people to understand words’ meaning). The former refers to the findings that abstract concepts lead to focusing attention primarily on events, social aspect of situation, while with concrete concepts attention focuses mainly on object or physical background of situation (Barsalou & Wiemer-Hastings, 2005; Barsalou, Dutriaux, & Scheepers, 2018). The latter refers to a recent notion introduced by Borghi et al. (2018; 2019), which consists of the awareness that our concepts are not sufficiently clear and detailed, and of the subsequent need to actively ask information from others to fill our knowledge gaps.

*Inner experience.*
As regards inner experience, we introduced dimensions of arousal, valence, interoception, and metacognition. Some authors considered emotionality, in terms of both arousal (ARO) and valence (VAL), as more crucial for abstract concepts than concrete concepts (Vigliocco et al., 2013; Vigliocco et al., 2014). Others underlined the importance of considering interoception (INT; i.e., awareness of inner body states) as an additional perceptual modality, likely more linked to abstract than concrete concepts (Connell & Lynott, 2018). Finally, the role of metacognition (META; i.e., cognition about our own cognitive processes) has been recently discussed in relation to abstract concepts processing (Borghi et al., in press).

*Sensorimotor experience and mouth/hand involvement.*To test whether the involvement of sensorimotor experience varied between abstract and concrete concepts, we asked participants to rate the perceptual strength of the five perceptual modalities (VISION, TOUCH, HEARING, SMELL, TASTE; Connell & Lynott, 2012; Lynott & Connell, 2013) and the ease with which a human body can physically interact with word’s referent (Body-Object Interaction, BOI; Siakaluk, Pexman, Aguilera, Owen, & Sears, 2008; Tillotson, Siakaluk, & Pexman, 2008; Bennett, Burnett, Siakaluk, & Pexman, 2011; Pexman, Muraki, Sidhu, Siakaluk & Yap, 2019). Finally, we included the rating on to what extent each concept involves the use of mouth (MOUTH) and hand effector (HAND). Recent evidence has indeed shown that during abstract concepts processing the mouth motor system is activated, while processing of concrete concepts more involves the hand motor system (Ghio et al., 2013; Borghi & Zarcone, 2016; Barca, Mazzuca, & Borghi, 2017; Dreyer & Pulvermüller, 2018).
