# Supplementary material for: Is justice grounded? How expertise shapes conceptual representation of institutional concepts
Source: Psychol Res. 2021 Mar 7;86(8):2434–50. doi: 10.1007/s00426-021-01492-8 (PMC9674748; doi:10.1007/s00426-021-01492-8)
Supplement: Supplementary file 2 — Supplementary file2 (DOCX 24 KB) [file 426_2021_1492_MOESM2_ESM.docx]

**Supplementary materials 2**

Means, for each dimension, in function of the interaction *Group* (Law-group and Control-group) x *Category* (Institutional, Theoretical, Food, Artefact) in the leftmost panel, and of the interaction *Group* (Law-group and Control-group) x *Type of Institutional* (Pure and Meta) in the rightmost panel.

|  |  |  | Group x Category | |  |  | Group x Type of Institutional | |
| --- | --- | --- | --- | --- | --- | --- | --- | --- |
| ABS-CNR |  | Institutional  Theoretical  Food  Artefact | Law Group  4.2  3.9  6.9  6.8 | Control group  3.9  4.1  6.7  6.6 |  | Pure  Meta | Law Group  4.5  3.7 | Control Group  4.7  3.0 |
| IMG |  | Institutional  Theoretical  Food  Artefact | Law Group  4.2  4.0  6.7  6.7 | Control group  3.7  3.8  6.7  6.6 |  | Pure  Meta | Law Group  5.0  3.4 | Control Group  4.9  2.4 |
| CA |  | Institutional  Theoretical  Food  Artefact | Law Group  5.5  4.1  5.1  5.4 | Control group  4.5  4.6  6.5  6.4 |  | Pure  Meta | Law Group  5.8  5.4 | Control Group  5.4  4.0 |
| FAM |  | Institutional  Theoretical  Food  Artefact | Law Group  6.3  4.6  5.8  5.7 | Control group  5.6  5.4  6.7  6.6 |  | Pure  Meta | Law Group  6.2  6.4 | Control Group  5.5  5.7 |
| AoA |  | Institutional  Theoretical  Food  Artefact | Law Group  4.2  4.2  2.1  2.3 | Control group  5.0  4.3  2.1  2.6 |  | Pure  Meta | Law Group  4.0  4.1 | Control Group  4.9  5.3 |
| MoA |  | Institutional  Theoretical  Food  Artefact | Law Group  4.8  4.7  2.0  2.3 | Control group  5.6  5.3  1.9  2.3 |  | Pure  Meta | Law Group  5.3  4.8 | Control Group  5.4  6.1 |
| SOC |  | Institutional  Theoretical  Food  Artefact | Law Group  5.9  2.7  1.9  2.7 | Control group  5.4  2.9  2.6  3.3 |  | Pure  Meta | Law Group  6.2  6.2 | Control Group  5.6  5.5 |
| MESO |  | Institutional  Theoretical  Food  Artefact | Law Group  3.2  3.2  1.3  1.5 | Control group  3.1  3.2  1.1  1.2 |  | Pure  Meta | Law Group  3.0  3.4 | Control Group  2.9  3.4 |
| ARO |  | Institutional  Theoretical  Food  Artefact | Law Group  4.2  2.5  2.3  2.7 | Control group  3.8  2.8  3.1  3.0 |  | Pure  Meta | Law Group  3.7  5.1 | Control Group  3.4  4.4 |
| VAL |  | Institutional  Theoretical  Food  Artefact | Law Group  5.1  4.3  5.0  4.5 | Control group  4.0  4.0  4.4  5.1 |  | Pure  Meta | Law Group  5.2  5.3 | Control Group  3.8  4.6 |
| INT |  | Institutional  Theoretical  Food  Artefact | Law Group  4.0  3.2  3.2  3.1 | Control group  4.0  3.0  3.2  3.1 |  | Pure  Meta | Law Group  3.4  4.4 | Control Group  3.5  4.2 |
| META |  | Institutional  Theoretical  Food  Artefact | Law Group  4.8  3.4  2.3  3.0 | Control group  4.7  3.8  3.0  3.7 |  | Pure  Meta | Law Group  4.6  5.1 | Control Group  4.2  5.1 |
| VIS |  | Institutional  Theoretical  Food  Artefact | Law Group  4.9  4.6  5.9  6.1 | Control group  3.9  4.3  5.9  6.0 |  | Pure  Meta | Law Group  5.9  4.3 | Control Group  4.6  3.3 |
| HEA |  | Institutional  Theoretical  Food  Artefact | Law Group  4.0  2.6  1.7  3.1 | Control group  3.0  2.3  1.7  2.9 |  | Pure  Meta | Law Group  4.1  4.1 | Control Group  2.8  2.8 |
| TOU |  | Institutional  Theoretical  Food  Artefact | Law Group  2.4  2.7  5.2  5.0 | Control group  1.6  2.6  4.5  4.6 |  | Pure  Meta | Law Group  2.2  2.1 | Control Group  1.5  1.4 |
| TAS |  | Institutional  Theoretical  Food  Artefact | Law Group  1.4  1.4  6.1  1.6 | Control group  1.2  1.3  5.8  1.6 |  | Pure  Meta | Law Group  1.1  1.2 | Control Group  1.0  1.0 |
| SME |  | Institutional  Theoretical  Food  Artefact | Law Group  1.6  1.6  5.3  2.4 | Control group  1.3  1.5  4.4  2.1 |  | Pure  Meta | Law Group  1.5  1.5 | Control Group  1.2  1.2 |
| BOI |  | Institutional  Theoretical  Food  Artefact | Law Group  3.7  3.3  1.7  2.0 | Control group  4.4  4.0  1.8  2.1 |  | Pure  Meta | Law Group  3.4  3.7 | Control Group  4.5  4.5 |
| MOUTH |  | Institutional  Theoretical  Food  Artefact | Law Group  4.2  3.0  5.0  2.9 | Control group  3.6  2.6  5.1  2.7 |  | Pure  Meta | Law Group  4.4  4.2 | Control Group  3.7  3.3 |
| HAND |  | Institutional  Theoretical  Food  Artefact | Law Group  2.7  2.8  4.4  5.0 | Control group  3.1  3.0  4.6  4.6 |  | Pure  Meta | Law Group  2.8  2.2 | Control Group  3.0  2.8 |
